# UNUSUAL PRESENTATION OF AN UNCOMMON LUNG MALIGNANCY

**DOI:** 10.4103/0970-2113.59596

**Published:** 2008

**Authors:** K Ramaraju, B Aggarwal, R Kulsrestha, SK Chhabra

**Affiliations:** 1 Department of Cardiorespiratory Physiology, Clinical Research Centre, University of Delhi, Delhi - 110 007; 2 Department of Pathology, Vallabhbhai Patel Chest Institute, University of Delhi, Delhi - 110 007

**Keywords:** Lung cancer, Combined carcinoma

## Abstract

Combined type of small cell lung carcinoma (SCLC) is a rare malignancy of the lung that is usually peripheral and diagnosed after resection. We report an unusual case of centrally located combined SCLC with squamous cell component that was diagnosed on endobronchial lung biopsy.

## INTRODUCTION

Small cell lung carcinoma (SCLC) accounting for 20-25% of all lung cancers, remains one of the most frequently diagnosed cancers and the most common cause of cancer deaths worldwide^1^,^2^. Combined type has been reported to occur in 1 – 3% of all cases of SCLC. These tumours consist of SCLC with a component of squamous cell carcinoma and/ or adenocarcinoma^3^. The rarity and heterogeneity of the tumour make the identification of combined SCLC difficult. Most are peripheral and diagnosed after resection. We report an unusual case of centrally located combined SCLC with squamous cell component that was diagnosed on endobronchial lung biopsy.

## CASE REPORT

**Fig. 1:**
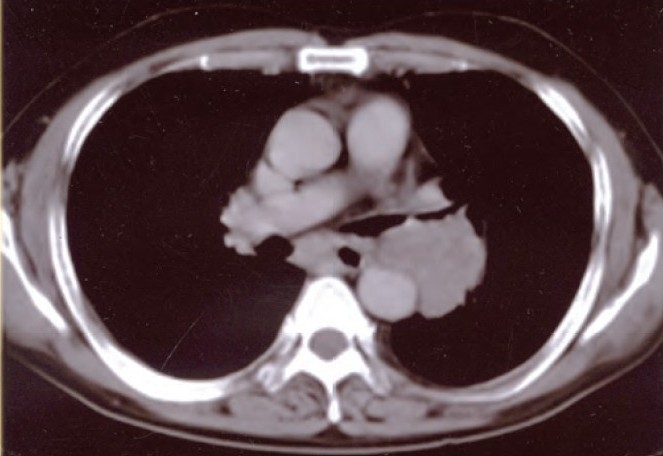
Contrast enhanced computed tomography scan of the chest showing the left hilar mass compressing the left main bronchus with enlarged subcarinal lymph nodes.

A 65 year-old male patient, current smoker (50 pack years) presented with complaints of dry cough and breathlessness for the past two months. He was also on treatment for hypertension for the last 3 years. Physical examination revealed no pallor, clubbing or lymphadenopathy. Examination of the respiratory system was not remarkable. The complete blood counts, electrocardiograph, and liver and kidney function tests (biochemistry) were within normal limits. The chest radiograph postero-anterior (PA) view taken one month apart showed a progressing mass lesion in the left paratracheal and parahilar region. Contrast enhanced computed tomography scan of the chest revealed a mass lesion with peripheral enhancement in the left hilum compressing the left main bronchus and encroaching on the wall of the descending aorta (Fig. 1). Subcarnial lymph nodes were enlarged. Spirometry showed moderately severe airflow limitation with FEV1/FVC of 68%, forced expiratory volume in one second (FEV_1_) of 56% predicted and forced vital capacity (FVC) of 67% predicted. Change after bronchodilator was not significant. Fibreoptic bronchoscopy under local anesthesia revealed extramural compression superomedially in the left main bronchus. A smooth surfaced growth was seen just beyond the compression. The overlying mucosa was hyperemic and the growth bled on touch. Biopsy was taken.

**Fig. 2:**
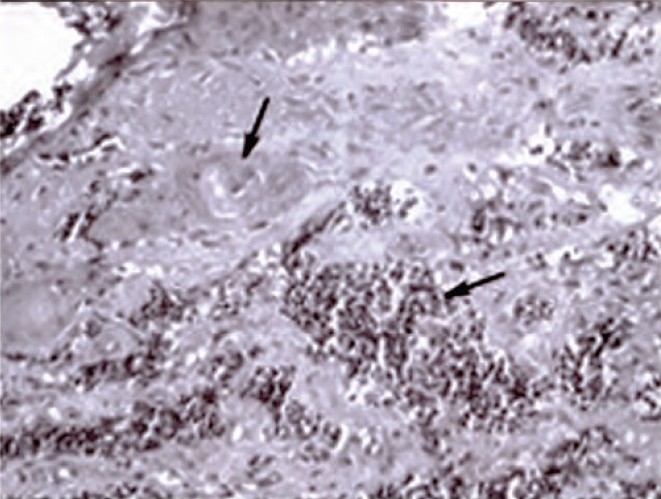
Postbronchoscopy sputum smear showing dual cell population. Papanicolau stain ×400

Bronchial aspirate did not show any atypical cells on microscopic examination However, post-bronchoscopy sputum showed a cluster of small atypical cells with dark blue hyperchromatic nuclei, nuclear overlapping and scant cytoplasm. Occasional singly arranged atypical keratinized squamous cells having irregular, angulated cytoplasmic margins and pyknotic nuclei were also seen (Fig 2). The picture suggested the presence of two malignant cell populations — small cell and squamous.

**Fig 3:**
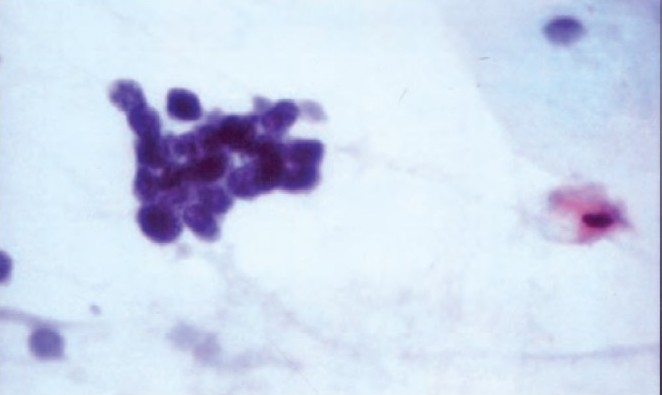
H & E (10x) stained microphotographs showing populations of small cell lung carcinoma and squamous cell carcinoma (arrows).

Histopathologic examination of the transbronchial lung biopsy specimen showed two cell populations with atypia (Fig 3). Superficial well-differentiated squamous cell carcinoma component with squamous epithelial pearl formation was identified. In deeper tissue an additional component of small cell carcinoma comprising of lymphoblast-like cells with large hyperchromatic nuclei, nuclear pleomorphism, nuclear overlap, increased atypical mitosis and scant amount of indistinct cytoplasm was seen. The alternative diagnosis for this component was Lymphoma.

**Fig. 4:**
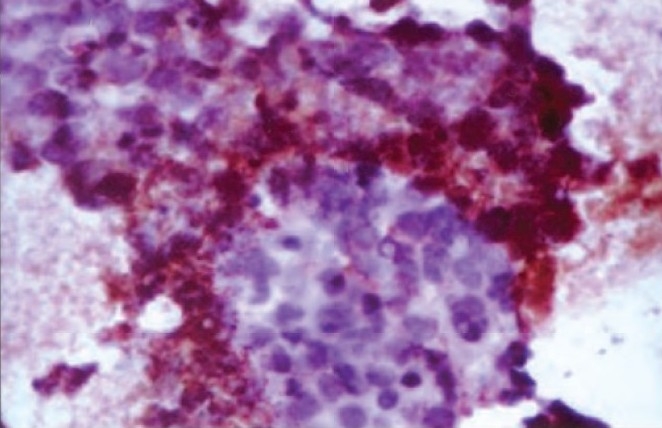
Synaptophysin positivity of small cell component on immunohistochemistry ×400

To confirm the diagnosis, immunohistochemistry was performed using a panel of antibodies (Dako, Carpinteria, CA, USA) to Chromogranin A, Synaptophysin and Common Leucocyte Antigen CD-45 (Clone: 2B11 & PD7/26). The slides were stained using streptavidin – biotin kit (DakoCytomation LSAB2 system -HRP). Positivity for Synaptophysin and CLA negativity were diagnostic for small cell carcinoma and ruled out the possibility of lymphoma (Fig 4).

A diagnosis of combined small cell lung carcinoma was thus made. The patient was explained the prognosis and referred for chemotherapy as surgical resection was ruled out due to the central location of the tumour and its close proximity to the aorta.

## DISCUSSION

SCLC is classified as a separate subtype of lung carcinoma because of its clinical distinction, natural history and different growth characteristics. According to the World Health Organization (WHO) criteria with modifications by the Pathology committee of the International Association for the Study of Lung Cancer (IASLC), three subtypes of SCLC have been recognized: pure (classic), mixed and ombined^4^. Mixed SCLC are tumours with a mixture of both SCLC and large cell carcinoma. Combined type SCLC has been estimated to comprise 1 – 3% of all types of chemonaive SCLC^4^,^5^. These tumors contain elements of SCLC mixed with a component of squamous cell carcinoma and/or adenocarcinoma^5^,^6^. The low incidence rate and the usually small and inadequate specimen obtained by bronchoscopic biopsy make it difficult to diagnose combined SCLC preoperatively^7^. Hage et al^8^ assessed 2115 patients with bronchogenic carcinoma and found that only 1.2% had combined SCLC. In none of the patients was the correct diagnosis established before operation. Most of the combined SCLC had stage 1 disease and surgical resection yielded a cumulative five-year survival of 31% while among those with stage 2 and 3 disease there were no survivors at five years. These outcomes were similar to those in patients with pure SCLC.

The clinical characteristics and course of the combined subtype of SCLC patients are similar to patients with other subtypes of SCLC^8^,^9^. Patients with combined SCLC have a higher incidence of peripheral lesions^9^. The central location of the tumour in the present case was unusual. Combined SCLC in the majority of cases reported has been a postoperative diagnosis using microscopic examination of the surgical resected specimens^8^–^10^. Rare cases of combined SCLC have however been diagnosed using a transbronchial lung biopsy (TBLB) or mediastinoscopic biopsy^9^,^10^. In the present case, the diagnosis of combined SCLC was established by histopathologic examination, including immunohistochemistry of the bronchoscopic biopsy specimen. While combined SCLC is as such rare, the atypical central location as in the present case is even rarer.
